# Supplementary material for: Complexome profiling of the Chlamydomonas psb28 mutant reveals TEF5 as an early PSII assembly factor
Source: Plant Cell. 2025 Mar 17;37(6):koaf055. doi: 10.1093/plcell/koaf055 (PMC12164590; doi:10.1093/plcell/koaf055)
Supplement: koaf055_Supplementary_Data [file koaf055_supplementary_data.zip › psb28_interactive.zip!/plots/intensity/qualityPlots/peptides/LCI5_Cre10.g436550.t1.2.html]

### Cre10.g436550.t1.2

Trivial name: LCI5  
SeqCoverage [median]: 39.70  
Mass: 32.31 kDa  
  
 

| Sequence | MissedCleavage | Mass [Da] | wtIntensity | muIntensity | Score |
| --- | --- | --- | --- | --- | --- |
| DAAPASSAPAR | 0 | 1012.49 | 3706.03 | 1731.81 | 101.65 |
| ESSTATVQASR | 0 | 1135.55 | 417.72 | 0.00 | 91.58 |
| QELESLR | 0 | 873.46 | 745.97 | 1352.07 | 91.63 |
| SNSPAPASSAPAPAR | 0 | 1379.68 | 2145.37 | 1553.00 | 78.32 |
| SSSPAPASSAPAPAR | 0 | 1352.67 | 6970.78 | 5706.00 | 129.34 |
| TALPADWRK | 1 | 1056.57 | 257.88 | 0.00 | 53.33 |
|  |  |  | 14243.7 | 10342.9 |  |
